# The Effects of a Single-Session Virtual Rumination Intervention to Enhance Cognitive Functioning in Veterans With Subjective Cognitive Symptoms: Multimethod Pilot Study

**DOI:** 10.2196/48525

**Published:** 2024-04-12

**Authors:** Tara Austin, Jennifer Smith, Borsika Rabin, Laurie Lindamer, James Pittman, Staley Justice, Elizabeth W Twamley, Crystal Lantrip

**Affiliations:** 1 Research Service VA San Diego Healthcare System San Diego United States; 2 Center of Excellence for Stress and Mental Health VA San Diego Healthcare System San Diego, CA United States; 3 The VA Center of Excellence for Research on Returning War Veterans Waco, TX United States; 4 Herbert Wertheim School of Public Health and Human Longevity Science University of California San Diego San Diego, CA United States; 5 UC San Diego Altman Clinical and Translational Research Center Dissemination and Implementation Science Center University of California San Diego San Diego, CA United States; 6 Department of Psychiatry University of California San Diego San Diego, CA United States; 7 Department of Psychology and Neuroscience Baylor University Waco, TX United States

**Keywords:** army, cognition, cognitive, emotion regulation, memory symptoms, memory, military, rumination, subjective cognitive decline, telehealth, telemedicine, veteran, worry

## Abstract

**Background:**

Subjective cognitive concerns (SCCs) entail perceived difficulties in thinking or memory, often reported without substantial objective evidence of cognitive impairment. These concerns are prevalent among individuals with a history of brain injuries, neurological conditions, or chronic illnesses, contributing to both psychological distress and functional limitations. They are increasingly considered to be a risk factor for future objective decline. A considerable number of individuals reporting SCCs also exhibit mental health symptoms, such as a history of trauma, depression, or anxiety. Interventions that address modifiable emotional and cognitive factors related to SCC could improve functioning and quality of life. Therefore, the use of emotion regulation strategies, especially those directed at minimizing rumination, could serve as a promising focus for interventions aimed at mitigating subjective cognitive concerns in veteran populations.

**Objective:**

This pilot study explored the feasibility, acceptability, and preliminary efficacy of a brief, 1-session emotion regulation intervention called “Worry Less, Remember More.” The Worry Less, Remember More intervention was designed to reduce rumination and improve subjective cognitive functioning in veterans with subjective cognitive changes (N=15).

**Methods:**

We randomized 15 veterans to either the active telehealth condition or waitlist control and completed the intervention. Participants were aged between 31 and 67 (mean 49.5, SD 10.1) years, and the sample was primarily male (12/15, 83%) and White (10/15, 67%). The most common diagnoses were posttraumatic stress disorder and depression. Following the intervention, veteran input was sought through semistructured interviews with a subset of 12 participants, examining feasibility, acceptability, and perceived efficacy. Preliminary efficacy was also measured using pre- and postintervention self-report measures.

**Results:**

Veterans reported that this intervention was acceptable, with 92% (11/12) of the sample reporting that they benefited from the intervention and would recommend the intervention to others with similar difficulties. Semistructured interviews revealed difficulties with feasibility, including problems with the remote consenting process, forgetting appointments, and needing additional strategies to remember to consistently use the interventions. The intervention improved self-reported cognitive symptoms on quantitative measures but did not improve self-reported rumination.

**Conclusions:**

This pilot study establishes the preliminary feasibility, acceptability, and efficacy of the Worry Less, Remember More intervention for veterans with subjective cognitive symptoms. Future iterations of the intervention may benefit from simplifying the electronic consent process, providing reminders for appointments, and incorporating compensatory cognitive strategies to assist with using the telehealth system, as well as applying the strategies learned in the intervention. While future research is needed with larger samples, including nonveteran populations, the intervention may also be a useful clinical tool to bridge care between neuropsychology clinics and mental health treatment.

## Introduction

Subjective cognitive concerns (SCCs) refer to perceived challenges in thinking or memory reported with either no evidence or only minimal objective evidence of cognitive impairment [1]. SCCs are common in people with histories of brain injuries, neurological illnesses, and other chronic illnesses and contribute to psychological distress and functional impairment [2-5]. More than 10% of US veterans aged 45 years or older report subjective cognitive symptoms [6]. Interest in SCCs has increased due to cognitive symptoms, such as “brain fog,” following the COVID-19 infection [7]. Research has found that SCCs are often related to potentially modifiable factors that influence cognition, such as mood, and many individuals with SCCs have mental health symptoms, including a history of trauma, depression, or anxiety [8-11]. A recent study in a neuropsychology clinic found that childhood trauma predicted SCCs and that this relationship was mediated by a ruminative thought style [12]. Emotion dysregulation, including rumination, contributes to and maintains psychopathology as well as cognitive dysfunction, particularly in those with affective illnesses [13] and has been implicated in the maintenance of neuropsychiatric illness [14,15]. Rumination, or the tendency to passively and persistently dwell on negative or problematic aspects of life, and the ruminative thought process, which includes the act of focusing on the potential causes and outcomes of the negative or problematic aspects of life, are forms of emotional dysregulation, which are patterns of emotional experience or expression that interfere with goal-directed activity [14]. Ineffective coping strategies perpetuate emotion dysregulation and, thereby, depression and other psychological disorders [16,17].

Emotion regulation approaches vary in their impact on both emotional health (eg, depression relapse and severity) and cognitive functioning. Less effective regulation strategies, such as rumination (eg, perseverative thought processes focusing on negative content) [18] and suppression (inhibiting the outward signs of inner feelings), are suggested to be cognitively taxing, thus diminishing cognitive resources [19]. Rumination and suppression have been associated with poor health and negative psychological outcomes in the general population as well as in veteran populations [20]. In veterans, rumination has been found to moderate the association between posttraumatic stress disorder (PTSD) or depression and risky behavior [21] and between moral injury and negative mental health symptoms [22]. Rumination is also associated with sleep problems in veterans with PTSD and depression [22]. There is also preliminary evidence that rumination may moderate the relationship between attentional difficulties and PTSD symptoms [23].

As such, emotion regulation strategy use, particularly strategies aimed at reducing rumination, may be a good target for intervention to reduce SCCs in veteran populations. In order to address these difficulties, we created a 1-session treatment called “Worry Less, Remember More,” integrating elements from Watkins’s [24] rumination-focused cognitive-behavioral therapy for depression and Gilbert’s [25] compassion-focused therapy (Multimedia Appendix 1 [24,26,27]). The psychoeducation portion consisted of concepts from evolutionary psychology as described by Gilbert [25], including an evolutionarily adapted attentional bias toward negative information, information about emotional regulation systems and their responses to trauma and stress, and attention as a limited resource that can be redirected. We used Watkins’s [24] 12-session rumination-focused cognitive-behavioral therapy for depression to provide specific examples of rumination, purposes of rumination, and 3 short intervention exercises. The session was designed to be delivered through telehealth, as there is evidence that telehealth and web-based interventions are feasible, acceptable, and efficacious in bridging care and supplementing existing mental health treatment [28,29], including in populations with cognitive difficulties [30].

In addition to establishing the feasibility and efficacy of emotion regulation interventions to improve subjective cognitive functioning, it is also important to establish the acceptability of the intervention to individuals with SCCs to increase treatment engagement, compliance, and completion [31]. For this study, we operationalized feasibility (including demand, implementation, practicality, and integration into existing systems) and acceptability according to Bowen et al [32], Pearson et al [33], and Sekhon et al [34]. The goal of this study was to establish the feasibility, acceptability, and preliminary efficacy of a 1-session, rumination-focused intervention for veterans with SCCs compared to a waitlist control condition.

## Methods

### Ethical Considerations

This study was approved by the Central Texas Veterans Healthcare System Institutional Review Board (IRB number 00697). All participants were informed of the purpose of the study, as well as the possibility of dropping out at any time, and signed written informed consent before participation. All included study data are deidentified, and all quotes are anonymous and have been carefully reviewed to have all potentially identifiable data removed. Participants were paid US $40 per visit (US $120 in total).

### Recruitment

Veterans were recruited from the Veterans Administration (VA) neuropsychology specialty clinics, primary care clinics, and mental health clinics. The majority of veterans who participated were referred by mental health providers, though some veterans self-referred through flyers placed on the medical campus.

Inclusion criteria included verbal endorsement of cognitive difficulties and veteran status. Exclusion criteria included (1) a score of <23 on the Montreal Cognitive Assessment (MoCA); (2) current substance abuse; or (3) a diagnosis of serious mental illness, such as schizophrenia or psychotic disorders. We screened 49 veterans for eligibility, of whom 9 were self-referred and 40 were referred by mental health providers. Of the 49 veterans referred, 23 did not return to complete informed consent, 4 declined to participate, 2 had MoCA scores <23, another 2 were ineligible due to substance use, and 1 was ineligible due to a diagnosis of severe mental illness. Thus, a total of 17 veterans were enrolled and randomized to either the intervention or waitlist control condition before baseline testing. Of the veterans randomized, 15 attended the first visit (baseline testing and intervention for the intervention group; baseline testing only for the waitlist control group), and 12 attended both the initial assessment and follow-up testing. The 12 participants who completed the intervention also completed quantitative assessments and qualitative interviews at the 8-week follow-up. All study procedures (including screening, consenting, assessment, and intervention) were delivered remotely using VA video telehealth software.

### Sample Characteristics

Participants’ were aged between 31 and 67 (mean 49.5, SD 10.1) years (Table 1), and the sample was primarily male (12/15, 83%) and White (10/15, 67%). The most common diagnoses were PTSD and depression. There were no significant differences between the full sample and the qualitative interview participant subset on age, gender, race or ethnicity, rates of PTSD or sleep difficulties, or the percentage of participants who completed all study visits. The qualitative interview group had a higher percentage of brain injuries and lower rates of depression.

**Table 1:** Demographic information, computerized patient record system (CPRS) diagnoses, and baseline self-report measures for study participants. The percentages might not add up to 100% due to rounding off.

Demographic			Intervention group (n=7)		Waitlist control group (n=8)		Qualitative sample subset (n=12)
Participants who completed the study, n (%)			6 (86)		N/A^a^		10 (83)
Age (years), mean (SD)			51.7 (11.5)		49.6 (8.7)		49.4 (10.1)
Gender (male), n (%)			5 (71)		7 (88)		10 (83)
**Self-reported race, n (%)**							
	Asian	0 (0)		1 (13)		0 (0)	
	White	5 (71)		6 (75)		8 (67)	
	Black	2 (29)		1 (13)		4 (33)	
**Self-reported ethnicity, n (%)**							
	Hispanic	1 (14)		1 (13)		1 (8)	
**CPRS diagnoses of interest, n (%)**							
	PTSD^b^	3 (42)		3 (38)		6 (50)	
	Depression	4 (57)		3 (38)		4 (33)	
	Sleep	1 (14)		2 (25)		3 (25)	
	ADHD^c^	1 (14)		0 (0)		1 (8)	
	Traumatic brain injury	2 (28)		2 (25)		4 (33)	
**Baseline self-report measures, mean (SD)**							
	BDI-II^d^	31.55 (10.54)		34 (8.83)		N/A	
	BAI^e^	20.75 (5.11)		17.36 (5.33)		N/A	
	RRS-SF^f^	19.6 (3.67)		23.33 (6.15)		N/A	
	BRIEF-A^g^	96 (26.7)		82.75 (30.96)		N/A	
	NSI^h^	44 (12.51)		37.38 (9.02)		N/A	
	Rivermead^i^	42.33 (8.96)		29.44 (5.71)		N/A	

^a^N/A: not applicable.

^b^PTSD: posttraumatic stress disorder.

^c^ADHD: Attention deficit hyperactivity disorder.

^d^BDI-II: Beck Depression Inventory-Second Edition.

^e^BAI: Beck Anxiety Inventory.

^f^RRS-SF: Ruminative Response Scale and Perseverative Thinking Questionnaire-Short Form.

^g^BRIEF-A: Behavior Rating Inventory of Executive Functioning-Adult.

^h^NSI: Neurobehavioral Symptom Inventory.

^i^Indicates statistically significant difference between groups (*P*<.05).

### Study Procedure

The pilot study was a 1-visit randomized controlled trial with 2 follow-up visits to gather outcome data. All study visits were conducted remotely, using the VA telehealth system. Before the start of the study, participants were randomized to either an intervention or waitlist control condition. At the first visit, participants in both conditions completed a comprehensive preintervention self-report battery. Immediately following the initial baseline measures, those randomized to the intervention condition then participated in a 30-minute psychoeducation and rumination-focused intervention, whereas those in the waitlist control condition were excused. Then, 8 weeks later, both groups completed follow-up behavioral measures, and the waitlist control group received the intervention. Both groups completed follow-up behavioral measures at the third visit, approximately 8 weeks after the second visit.

### Measures

Measures administered included the Beck Depression Inventory-Second Edition (BDI-II) [35], the Beck Anxiety Inventory (BAI) [36], the Ruminative Response Scale and Perseverative Thinking Questionnaire-Short Form (RRS-SF) to assess rumination [37], the Behavior Rating Inventory of Executive Functioning-Adult (BRIEF-A) self-report [38], the Neurobehavioral Symptom Inventory (NSI) [39], and the Rivermead Post-Concussion Symptoms Questionnaire [40]. Raw scores from the BRIEF-A and cognitive items from the Rivermead and NSI were summed into a cognitive index score for each participant.

### Statistical Analysis

Quantitative analyses were performed in R (R Core Team) using statistical packages and the original code [41]. The data were checked for normality and outlier values; no corrections were needed. When <10% of data were missing at random, missing values were estimated and imputed using a random forest–based imputation, which has been shown to be appropriate for imputing mixed continuous or categorical data even in the presence of potential interactions and nonlinearity [42]. Differences in pre-post outcome measures (rumination and cognitive composite score) were assessed using repeated measures ANOVA with group as a between subject factor and time point (pre or post) and test as within-subject factors. Significant ANOVAs were followed up with 2-tailed *t* tests. Post hoc power analysis showed that with our sample size, we were only 15% powered to find an effect similar to previous single-session studies, which found small effects on emotional functioning (*d*=0.10-0.30). Based on the effect sizes in these studies, 139 participants per group would be needed for an α=.05 and a power of 0.80. As such, the purpose of this study is only to determine preliminary efficacy.

### Qualitative Data Collection and Analysis

We created an interview guide based on acceptability and feasibility concepts from Sekhon et al [34] and Bowen et al [32]. One portion of the interview guide consisted of 2 questions asked of all participants (“would you recommend this intervention to others with thinking difficulties?” and “did you feel you benefited from this intervention?”). The second portion of the interview guide contained flexible questions and prompts related to intervention cohesiveness, perceived effectiveness, usability, feasibility, and preferences. Example prompts from the interview guides are included in Table 2.

Interviews were conducted by 2 researchers at least 1 month after the second visit, and each interview was conducted by a researcher not involved in intervention delivery for each participant. Interviews lasted 20-45 minutes and were recorded and transcribed verbatim by the primary researcher (TA). Following each interview, a memo was written describing the conversation, themes observed, and experience of the interviewer. During the interview and transcription, responses were put under the construct addressed. If a response did not appear to address a construct, it was written at the bottom of the interview form. Following transcription, these responses were read and coded according to pre-established codes. Pre-established codes and themes were identified through discussions with participants before initiating the formal qualitative portion of the project. Additionally, themes that appeared in both the data memos before the transcription process and after all interviews were completed were coded. All interviews were read and coded for evidence of emerging codes. A secondary coder met to review and discuss the assigned codes. Discrepancies between coders were resolved by discussion.

**Table 2:** Quotes about veterans’ experiences of the intervention.

Constructs and examples of interview questions		Veterans’ responses
**Participant satisfaction**		
	Would you recommend this program to other Veterans?	92% (11/12) said “yes”
	Do you feel you benefited from this intervention?	92% (11/12) said “yes”
**Perceived difficulties: preintervention**		
	What lead you to sign up for this intervention? Did you have any specific goals going into the intervention or areas you wanted help with? What were some areas of difficulty before the intervention?	“It takes me longer to complete tasks,…. Longer and longer to complete tasks” “I’m having [a] lot of issues with concentration staying on task, forgetfulness, when I was younger I had ADD [attention deficit disorder]. I didn’t know about adult ADD and it is something you don’t grow out of?” “To improve memory- that was the hope, but no expectations” “[I] got out of the military, can’t concentrate. I quit things, can’t wrap my head around them” “Hard time coming up with my own thoughts” “Don’t remember things” “Walk from one room to another room, go in the room and forget why I’m there”
**Initial expectations**		
	What were your initial expectations of the intervention? How did your referring provider explain the intervention to you?	“It was supposed to help with my memory, correct? It sounds like a cliché but I didn’t really have any expectations.” “It was about memory loss, wasn’t it? I guess thinking, maybe some ideas on how to improve my memory.” “Well, they were doing a study for Veterans who were having cognitive issues.” “I didn’t really know what to expect.” “I originally from what I heard and read into, I thought it would be giving me some skills to help combat the cognitive decline I have had over the past of the few years, not mentally a decline, but a slowing of my thought process.” “Not really sure, I didn’t really, didn’t really have much. I guess to help others.”
**Perceived effectiveness**		
	Have any specific things in your life changed? Do you feel you benefited from the intervention? Has another (family members, coworkers, or medical team) noticed changes in either your mood or cognitive functioning?	“The wife has noticed my attitude and like that I have helped and she thought my participation was positive” “My wife has picked up the fact I am a bit more in tune, especially if we are in a public situation.” “My wife has mentioned [me] being in a better mood. Getting up and doing more things. Now being a bit more detail focused” “I do. It’s not like taking a pill and automatically fixed. It takes work on my part. Still can’t get over that hump in real life.”
**Barriers to participation or using strategies**		
	Were there any barriers to participation?	“If they could email appointment reminders, like a hard copy, just as a physical reminder. And y’all may have done that, I don’t know” “I just… I just hardly ever have my ringer on on my phone. I have problems following through with stuff like that” “I have never actually been tested, but I think I have a learning disability, but I think I have dyslexia. Yes, that’s one of the barriers. Had to read the questions a couple different times.” “Technical difficulties with VVC [VA video connect system], took about an hour or so for the questions.”
**Target population**		
	Who do you think would benefit from a program like this?	“Combat veterans for sure” “Would be helpful to do post-deployment”

## Results

### Quantitative Findings

There were no significant differences between groups on preintervention measures of rumination, perseverative thinking, or mood. There was a significant difference on one measure of neuropsychiatric symptoms, with higher reported cognitive difficulties on the NSI in the treatment group (Table 1). Both groups reported high levels of neuropsychiatric symptoms, rumination, and perseverative thinking, as well as severe symptoms of depression and moderate symptoms of anxiety.

### Acceptability

A total of 92% (11/12) of participants reported they benefited from the intervention and would recommend the intervention to other veterans with similar difficulties. The 1 veteran who reported they did not benefit directly from the program reported benefiting somewhat from the intervention, as it was able to provide a referral to more intensive cognitive rehabilitation treatment.

### Efficacy

When examining the postintervention outcome measures (rumination and the cognitive composite index), there was a significant interaction between time and group for the cognitive composite index (*F*_1,13_=5.97; *P*=.03), with significant improvement in the intervention group and not the control group (small effect *d*=0.10). There was not a significant interaction between time and group on measures of self-reported rumination.

### Qualitative Findings

#### Preintervention Expectations and the Motivation to Participate

Veterans reported few, if any, expectations for the intervention, with comments like “I didn’t really know what to expect” or reported general expectations about improving cognition, such as “It was supposed to help with my memory, correct?” (Table 2 contains participant responses). A total of 92% (11/12) veterans reported that their primary motivation to start the intervention was to have tools to help with their daily difficulties with cognition. One veteran reported that his primary motivation to participate was to improve future treatments for other veterans experiencing cognitive problems.

#### Acceptability

When asked about recommending the intervention to others with similar difficulties, several veterans (n=4 with similar comments) reported that the intervention “would be helpful to do postdeployment” and should be given to all “combat veterans for sure” before the development of cognitive difficulties. The feedback on perceived effectiveness was generally positive, with most veterans reporting improvement in daily functioning. Notably, more veterans reported their spouse or partner had noticed improvement (n=6) compared to those who stated they had noticed improvement themselves (n=4).

#### Feasibility

A barrier to participation in the study was the required remote consenting procedures. A total of 23 veterans expressed interest but did not return the consent form. Those who participated in the intervention reported that the remote consenting process was more difficult compared to previous in-person consenting for research participation. Another barrier to participation was forgetting appointments, and many participants had to be rescheduled multiple times due to forgetting appointments. Veterans were called the week of their appointment to remind them of their appointment; however, they requested additional reminders and reminders in digital modalities, such as “email appointment reminders, like a hard copy, just as a physical reminder.” Retention rates improved when the telehealth system started to send out SMS text message reminders of the appointment along with the email reminder.

Even though 92% (11/12) of participants said they recommended the intervention, only 2 out of the 12 veterans were able to describe and recall an exercise from the study, and several indicated that they forgot what the exercises were. Of the 10 veterans who did not remember the specifics of the intervention, 2 reported it was helpful to learn more about additional resources for both cognitive skills and treatments for mood, which they were able to pursue after the intervention. A veteran reported it was helpful as “this helped me put things into words and helps me understand. Sometimes you don’t know how to put things into words.” He reported that being able to explain both his cognitive difficulties and mood symptoms had improved communication with his family and members of his health care team. In contrast to the intervention exercises, the psychoeducation portion was noted as helpful in interviews with 10 veterans.

## Discussion

### Overview

We found the preliminary efficacy of a 1-session rumination-focused intervention to improve cognitive symptoms; however, there was no change in rumination, which was the proposed treatment target and mechanism. In qualitative interviews, veterans reported this intervention was acceptable and beneficial, as evidenced by over 92% 11/12) stating they benefited from the intervention and would recommend the intervention to others with similar difficulties. In addition to being acceptable to veterans, there was a high level of perceived effectiveness and intervention cohesiveness. Due to the discrepancy between self-reported improvement and the scores on the RRS-SF, further research may benefit from exploring the use of other rumination or perseverative thought measures.

The psychoeducation and brief intervention modules have potential utility in bridging care after neuropsychological or neurological evaluations and subsequent referrals to mental health treatment. Brief, internet-administered interventions have been shown to be effective in bridging care for mental health symptoms [28]. The brief interventions described in the module and handouts can then be used to build awareness while waiting to be seen by mental health clinicians or for existing mental health patients to understand how their treatment may improve their cognitive functioning. In these cases, the intervention can be incorporated into the feedback session and may yield additional “buy-in” from veterans to fully participate in mental health treatment. Due to the large number of veterans reporting SCC, telehealth-based intervention is an efficient and cost-effective way to meet these needs [43].

There are several limitations in this study, including the small sample size, which limited statistical analyses. Our pilot work suggests that veterans with subjective cognitive changes are amenable to psychological treatment and perceive benefit from a short, 1-session intervention. Further work with a larger sample size is needed to fully evaluate efficacy and whether the intervention described here should be implemented more broadly. Additionally, a larger sample size will allow further exploration of how changes in rumination may or may not mediate the relationship between SCCs and cognition. Further studies can then evaluate whether the psychoeducation about rumination is sufficient to fulfill the long-term goals of the intervention, namely, to bridge care following referral to mental health services and increase veteran buy-in for participation in psychological treatments to improve cognition. Further research in this area could also explore the use of a longer intervention as a standalone treatment.

### Conclusion

In conclusion, we found preliminary evidence for the feasibility, acceptability, and efficacy of a 1-session rumination-focused intervention for veterans with SCCs, which will benefit from continued evaluation of this intervention as well as comparison to routine clinical practice.

## Appendix

Multimedia Appendix 1 "Worry less, remember more" intervention and resources.

## Abbreviations

BAI: Beck Anxiety Inventory
BDI-II: Beck Depression Inventory-Second Edition
BRIEF-A: Behavior Rating Inventory of Executive Functioning-Adult
MoCA: Montreal Cognitive Assessment
NSI: Neurobehavioral Symptom Inventory
PTSD: posttraumatic stress disorder
RRS-SF: Ruminative Response Scale and Perseverative Thinking Questionnaire-Short Form
SCC: subjective cognitive concern
VA: Veterans Administration

## Data Availability

The data sets generated during or analyzed during this study are available from the corresponding author on reasonable request.
