# Supplementary material for: The Effects of a Single-Session Virtual Rumination Intervention to Enhance Cognitive Functioning in Veterans With Subjective Cognitive Symptoms: Multimethod Pilot Study
Source: JMIR Form Res. 2024 Apr 12;8:e48525. doi: 10.2196/48525 (PMC11053393; doi:10.2196/48525)
Supplement: Multimedia Appendix 1 [file formative_v8i1e48525_app1.docx]

APPENDIX A

**“Worry Less, Remember More” Intervention**

(Italics provide possible ways to explain concepts and do not need to be read or asked verbatim).

**Questions about areas of concern/personalizing the brief intervention:**

The goal of the questions is to get information to personalize the psychoeducation portion to specific difficulties of the individual:

*What are some difficulties that brought you to participate in our interventions?*

*What is the reason you think you have these cognitive problems (e.g., cause)? Is this something you’ve been told or read about?*

*What do you know about this condition or how it is relating to your current cognitive problems?*

*Is there anything else going on in your life that might also be contributing to your thinking difficulties?*

**Psychoeducation – “Tricky brain” based on the works of Paul Gilbert and Chris Irons:**

*There are many reasons for thinking difficulties including head injuries, (and stress from the injuries), difficulties sleeping, mood difficulties, trauma from the event or other events (PTSD), and pain. Our goal today is to help you understand common causes of thinking difficulties and some practical skills to help with current symptoms.*

*Before we talk about what might be contributing to your current difficulties, it is important to learn a bit more about our brain, including how it works and how it has changed, and will continue to change, in response to what has occurred in our life. Similar to how our bodies have adapted over the last thousands or millions of years, or brains have also adapted and grown in response to our environments.*

*One researcher, Paul Gilbert describes us having “an old brain… that we shared with many other animals walking this earth, and the anatomical structures of this part of the brain coordinate important yet ancient motivations (for basic survival like food and shelter, as well as relationships and status), behaviors (like flight, flight, and freeze), and emotions (e.g., anger, anxiety, and sadness”* (Irons, 2014).

*Similar to how adaptations in our physical body don’t always match our environment (such as cravings for fatty and sugary foods that would have kept our caveman ancestors full for a long time), some of the ways our brain has adapted are very helpful (yay creativity) and some are “trade-offs” or “glitches” that provided benefits but also costs.*

*As our brains are full of both useful adaptations as well as some evolutionary glitches, it can be helpful to think of our brains as “tricky” or not always adapted in a way that will help us interact most effectively with our environment. One such adaption that is not always helpful is our brains focus on the negative, or what either has gone wrong or could go wrong.*

**Psychoeducation – Neuroplasticity:**

*But the good news is that our brains are changing throughout our lives in response to what happens to us and how we engage with the world. The brain’s ability to change is referred to as neuroplasticity. Whatever we do repeatedly, whether helpful or unhelpful, will become more automatic.*

*While this might sound complicated, this is something we see in everyday life. (Ask if they ever learned a sport, musical instrument, or foreign language). When playing piano (or baseball, or another sport, or learning Spanish), at first it might have felt very difficult. Over time, it became easier.*

*This is also why coaches or teachers look for bad habits early on, these habits can also become more automatic over time. These principles apply not only to behaviors (such as learning a sport), but also our thoughts and feelings. While some of our default thinking patterns might be less helpful (because of our tricky brains!), proactively paying attention to your thoughts and behaviors can lead to meaningful change in your lives.*

**Psychoeducation - Rumination:**

*When someone has difficulties with their thinking, regardless of cause, they can start to experience more of what we call rumination. Examples of rumination include: dwelling on difficulties, getting stuck on events from your past, and becoming preoccupied with something and not being able to get it out of your mind. It can feel a bit like running on a hamster wheel, you’re expending a lot of energy but not really getting anywhere.*

*Although rumination is normal, too much rumination can become problematic, usually because it leads to thinking more about the cause of problems than the solutions. For example,* (insert example similar to their difficulties*).*

*Why might rumination be unhelpful?*

*Do you feel there are aspects in your life you ruminate on?*

*Have you noticed a difference in getting stuck on your thoughts since you started to have more thinking problems?*

*There are several ways to help improve rumination. These involve noticing our thinking and changing from being “cause” focused to “solution” focused.*

1. **The 2-minute rule (Addis & Martell, 2004)**

*Ruminating can often be confused with problem solving. One way to tell apart problem-solving and rumination is to use the two-minute rule. If you are problem-solving for more than two minutes with no progress or additional insight into the problem, it probably is rumination not problem solving! It can be helpful to label this “ruminating” and switch tasks and come back to the problem later. Later, when you find yourself thinking of the same problem, monitor for any progress, however small after two minutes and if not, switch tasks. This will help you develop a solution focused though thinking style.*

*When we are solution focused, we are using the part of our brain that helps us identify solutions, make good decisions, and use creativity (*point to frontal part of the brain on handout*). When we ruminate, we often are engaging the part of our brain that is more focused on looking for safety (*point to the limbic system*) than helping us engage creativity in our lives.*

1. **Ask “how” rather than “why” questions (Watkins, 2016)**

*Questions like, “Why did this happen to me?” rather than “how can I work with my current challenges” can contribute to rumination. This week, try to notice when you are asking “why” questions and try to turn them into “how” questions. Similar to the two-minute rule, asking “how” engages the frontal portion of our brain and can help us find solutions and engage creatively with our world.*

1. **Scheduled worry time (modified by McGowan & Behar, 2012)**

*One technique that is helpful when having attention or memory problems is to stop multitasking. If you talk on the phone while cooking, you are more likely to forget to put in an ingredient or forget to take something out of the oven! We might not think of our thoughts in the same way, but if we are ruminating or worrying while working, talking with others, and doing something important, we are also multitasking! If you are talking to (insert spouse, family member, friend, etc.) and you are worrying that you are going to forget what they are saying later, you are multitasking! This means you are paying less attention to what they are saying, more likely to be distracted, more likely to misunderstand something they are saying, and also, more likely to forget what they said later! To help multitask less, we can schedule time to worry, just in the same way we can schedule time to pay bills or go to dinner.*

*To do this, you schedule a regular time (same time, place, and length of time) to worry. When you worry outside of this time or become aware of worries, note your worry/thought in a notebook or paper, and then postpone your worry until your worry time.*

*While this might seem like an unusual idea, scheduling time for worrying is helpful for many reasons. One, you notice how often you might worry during the day, since you must notice these thoughts and save your worries for later. Often what feels like a lot of worries is the same one to two worries interfering multiple times throughout the day. During your worry time, since you are not multitasking, you can pay more attention to what you are worried about, and possibly find more solutions.*

1. **Exchanging avoidance behaviors to approach behaviors (Watkins, 2016)**

*One way rumination is unhelpful is that it often leads to avoidance. One way to counteract this is to identify areas of avoidance and replace them by approaching. An example of this is ruminating might be with the goal of “avoiding forgetting a conversation.” Replacing this thought with an approach could be “put down my phone when talking to someone.” Switching avoidance to approach is helpful for a few reasons. One, again, it helps develop a solution focused mindset. Two, it is easier to see achievement and progression in doing something than avoiding not doing something!*

| **Common Avoidance Behaviors** | **Common Approach Behaviors** |
| --- | --- |
| Ruminating | Asking people for support and help |
| Staying in bed | Being assertive |
| Putting off jobs or task at work or home | Trying new things |
| Avoiding people | Social contact |
| Avoiding promotions at work | Problem solving |
| Avoiding evaluation or judgment by others (e.g., tests, exams, interviews) | Scheduling enjoyable activities or activities that give you purpose |
| Avoiding conflict | Allowing oneself to experience feelings |
| Distraction (watching a lot of TV, video games) | Making plans and decisions |
| Reassurance seeking | Taking responsibility |

**Additional resources:**

Additional topics that may be beneficial for providers to address with individuals with subjective cognitive difficulties:

- Psychoeducation on evolutionary development of thought patterns and “tricky” brains
  - For more on this topic, see chapter 2 and 23 in Gilbert, P., & Simos, G. (Eds.). (2022). *Compassion focused therapy: Clinical practice and applications*. Routledge, chapter 4 in *CFT made simple book* and module 1 and 2 in Cattani, K., Griner, D., Erekson, D., Burlingame, G., Beecher, M., & Alldredge, C. (2021). *Compassion Focused Group Therapy for University Counseling Centers: A Clinician’s Guide.* Routledge.
- Additional information on rumination, such as ruminative thoughts as habits, rumination as a form of avoidance, and reasons for rumination (desire for seeking understanding and insight, planning and preparation, avoiding an “unwanted self,” preempting others’ criticism, controlling feelings)
  - For more on this topic see chapter 4 in Watkins, E.R. (2016). *Rumination-focused cognitive-behavioral therapy for depression.* Guilford Press, chapter 3 and 6 in Hayes & Smith *Get out of your mind and into your Life: New Harbinger Publications*

Individuals with subjective cognitive concerns seen in the study often had other potentially modifiable behavioral health factors that also likely contributed to reduced cognitive functioning. While not an exhaustive list by any means, the following resources have benefited us in our work with these patients and may be of use to either clinicians or when providing information to patients:

**Chronic Pain:**

Clinician:

- Louw, A., Puentedura, E. J., Diener, I., Zimney, K. J., & Cox, T. (2019). Pain neuroscience education: Which pain neuroscience education metaphor worked best?. *The South African journal of physiotherapy*, *75*(1), 1329. https://doi.org/10.4102/sajp.v75i1.1329
- Louw, A., Zimney, K., O’Hotto, C., & Hilton, S. (2016). The clinical application of teaching people about pain. *Physiotherapy theory and practice*, *32*(5), 385-395.
- Nijs, J., Wijma, A. J., Willaert, W., Huysmans, E., Mintken, P., Smeets, R., ... & Donaldson, M. (2020). Integrating motivational interviewing in pain neuroscience education for people with chronic pain: a practical guide for clinicians. *Physical therapy*, *100*(5), 846-859.
- Louw, A., Puentedura, E., Schmidt, S., & Zimney, K. (2018). *Pain neuroscience education: Teaching people about pain*. Orthopedic Physical Therapy Products.
- Moseley, G. L., & Butler, D. S. (2017). *Explain pain supercharged*. NOI.

Patient:

# Louw, A., Diener, I., Raymond, C., & Bohner, R. (2014). *Your Headache Isn’t All In Your Head Neuroscience Education for Patients with Headache Pain.* Orthopedic Physical Therapy Products

# Louw, A. (2013). *Why Do I Hurt? - A Patient Book About the Neuroscience of Pain.* Orthopedic Physical Therapy Products

# Louw, A., Flynn, T., & Puentedura, E (2015). *Everyone Has Back Pain - Neuroscience Education for Patients with Back Pain.* Orthopedic Physical Therapy Products

**Adjusting to chronic illness/changes in function:**

Patient:

- Sawatsky, J. (2017). *Dancing with Elephants: Mindfulness Training for those living with Dementia, chronic illness, or an aging brain*. Red Canoe Press.
- Bernhard, T. (2018). *How to be sick: A Buddhist inspired guide for the chronically ill and their caregivers.*Wisdom Publications.
- Kalanthi, P. (2016). *When breath meets Air.* Random House.
- Pausch, R. (2008). *The Last Lecture.* Two Roads.
- Sacks, O. (2015). *Gratitude.* Knopf Canada.
- Gawande, A. (2014). *Being Mortal.* Metropolitan Books.

**Sleep:**

Clinician:

- Mindfulness based tools to help with sleep in Chapter 11 in Khazan, I. (2019). *Biofeedback and Mindfulness in Everyday Life: Practical Solutions for Improving Your Health and Performance.* W.W. Norton & Company

Patient:

- Walker, M. (2017). *Why We Sleep: Unlocking the power of Sleep and Dreams*. Scribner.
- Winter, C.W. (2017). The Sleep Solution: Why Your Sleep is Broken and How to fix it. Berkley Press

**Stress:**

Clinician:

- Santorelli, S. (2010). *Heal thy Self: Lessons on Mindfulness in Medicine*. Harmony Publications.
- Lehrer, P., Woolfolk, R., & Sime, W. (2007). *Principles and Practice of Stress Management (Third Edition).* The Guilford Press.
- Steffen, P. R., Hedges, D., & Matheson, R. (2022). The Brain Is Adaptive Not Triune: How the Brain Responds to Threat, Challenge, and Change. *Frontiers in psychiatry*, *13*, 802606. https://doi.org/10.3389/fpsyt.2022.802606

Patient:

- Sapolsky, R. (2015). *Why Zebras Don’t Get Ulcers: The Acclaimed Guide to Stress, Stress-Related Disorders, and Coping (Third Edition).* Holt Paperbacks
- Mulligan, B. (2017). *The Dharma of Modern Mindfulness.* New Harbinger Publications.

**Handout or information for recommendations in reports or treatment plans:**

1. **The 2-minute rule^1^**

For the next few days, pay attention to when you start thinking about your current difficulties or frustrations. These are times when you might start ruminating. If you find yourself thinking about these topics for more than two minutes with no progress or additional insight into the problem, it probably is rumination not problem solving! It can be helpful to label this “ruminating” and switch tasks and come back to the problem later. Later, when you find yourself thinking of the same problem, monitor for any progress, however small after two minutes and if not, switch tasks. This will help you develop a solution focused though thinking style.

1. **Ask “how” rather than “why” questions^2^**

Questions like, “Why did this happen to me?” rather than “how can I work with my current challenges” can contribute to rumination. This week, try to notice when you are asking “why” questions and try to turn them into “how” questions.

1. **Scheduled worry time^3^**

The goal of scheduling worry time is to reduce multitasking by worrying while doing important things during the day! When you do this, you can aren’t able to pay attention fully to either the task or coming up with any insights or solutions to the things you are worried about. To help with this, you can schedule a specific time to worry.

Start by scheduling a regular time (same time, place, and length of time) to worry. When you worry outside of this time or become aware of worries, note your worry/thought in a notebook or paper, and then postpone your worry until your worry time. If you worry you will forget your worry, that’s okay! If you forget, is it really that important to worry about?

While this might seem like an unusual idea, scheduling time for worrying is helpful for many reasons. One, you notice how often you might worry during the day, since you must notice these thoughts and save your worries for later. Often what feels like a lot of worries is the same one to two worries interfering multiple times throughout the day. During your worry time, since you are not multitasking, you can pay more attention to what you are worried about, and possibly find more solutions.

1. **Exchanging avoidance behaviors to approach behaviors^3^**

One way rumination is unhelpful is that is can lead to avoidance. One way to counteract this is to identify areas of avoidance and replace them by approaching. An example of this is ruminating might be with the goal of “avoiding forgetting a conversation”. Replacing this thought with an approach could be “put down my phone when talking to someone”. Switching avoidance to approach is helpful for a few reasons. One, again, it helps develop a solution focused mindset. Two, it is easier to see achievement and progression in doing something than avoiding not doing something! This week, identify one or two behaviors in the avoidance column and see if you can switch them for one or two behaviors in the approach column.

| **Common Avoidance Behaviors** | **Common Approach Behaviors** |
| --- | --- |
| Ruminating | Asking people for support and help |
| Staying in bed | Being assertive |
| Putting off jobs or task at work or home | Trying new things |
| Avoiding people | Social contact |
| Avoiding promotions at work | Problem solving |
| Avoiding evaluation or judgment by others (e.g., tests, exams, interviews) | Scheduling enjoyable activities or activities that give you purpose |
| Avoiding conflict | Allowing oneself to experience feelings |
| Distraction (watching a lot of TV, videogames) | Making plans and decisions |
| Reassurance seeking | Taking responsibility |

^1^ Addis, M. E., & Martell, C. R. (2004). *Overcoming depression one step at a time: The new behavioral activation approach to getting your life back*. New Harbinger Publications, Incorporated.

^2^ Watkins, E.R. (2016). *Rumination-focused cognitive-behavioral therapy for depression.* Guilford Press.

^3^ McGowan, S. K., & Behar, E. (2013). A preliminary investigation of stimulus control training for worry: Effects on anxiety and insomnia. *Behavior Modification*, *37*(1), 90-112.
